# The Regulatory Role of CircAGGF1 in Myogenic Differentiation and Skeletal Muscle Development

**DOI:** 10.3390/ani15050708

**Published:** 2025-02-28

**Authors:** Wei Hei, Yuxuan Gong, Wenrun Cai, Ruotong Li, Jiayi Chen, Wanfeng Zhang, Mengting Ji, Meng Li, Yang Yang, Chunbo Cai, Xiaohong Guo, Bugao Li

**Affiliations:** College of Animal Science, Shanxi Agricultural University, Jinzhong 030801, China; heiwei1234@126.com (W.H.); gongyuxuan0408@163.com (Y.G.); 19834543032@163.com (W.C.); z20223512@stu.sxau.edu.cn (R.L.); 15735347824@163.com (J.C.); b20201040@stu.sxau.edu.cn (W.Z.); jimengting2864@163.com (M.J.); mengli@stu.sxau.edu.cn (M.L.); yangyangyh@stu.sxau.edu.cn (Y.Y.); caichunbo@stu.sxau.edu.cn (C.C.)

**Keywords:** circAGGF1, miR-199a-3p, Fgf7, C2C12, myogenesis, muscle fiber type

## Abstract

Circular RNA (circRNA) has a significant impact on the maturation of skeletal muscle, although their precise functions within this framework remain largely uncharted. This study presents an investigation of the regulatory effect of circAGGF1 on myogenesis in myoblasts, including the potential molecular mechanisms involved. It is revealed that circAGGF1 facilitates the differentiation of myoblasts into other states while simultaneously enhancing the manifestation of type I muscle fibers. In vivo investigations with mice revealed the promotion of skeletal muscle expansion and maturation by circAGGF1, bolstering its regenerative capacity. Mechanistically, circAGGF1 interacts with miR-199a-3p by acting as a sponge, promoting the subsequent expression of Fgf7. Furthermore, rescue experiments indicated a counteraction of the myogenesis induced by circAGGF1 overexpression by miR-199a-3p. To summarize, this research highlights the role played by circAGGF1 in the development of skeletal muscle, providing a valuable resource for enhancing our understanding of skeletal muscle biology.

## Simple Summary

Meat quality and skeletal muscle development are closely linked. Recent studies have highlighted the significant impact of circular RNAs (circRNAs) on skeletal muscle development, although their specific roles and regulatory mechanisms in this context remain largely elusive. This article describes a newly identified circRNA, circAGGF1, found in porcine skeletal muscle, leveraging previously acquired sequencing data. It was found that circAGGF1 promotes muscle differentiation via the circAGGF1/miR-199a-3p/Fgf7 axis in vitro. Furthermore, in vivo, circAGGF1 accelerates the regeneration and development of muscle. The mechanism of muscle development regulation via circRNA is given new insights by these results.

## 1. Introduction

Skeletal muscle serves as a crucial linchpin for bodily integrity, motion execution, and metabolic homeostasis in the mammalian anatomy. Its evolutionary blueprint entails a sophisticated cascade: it begins with the rousing of latent satellite cells, after which myoblasts proliferate and specialize, and ends with the synthesis of muscle fibers [1,2]. The quantity of muscle fibers remains stable in the embryonic phase, whereas after birth, skeletal muscle advances primarily through the augmentation in girth of individual fibers. Adult mammalian muscle fibers are categorized into four distinct myosin heavy chain (MyHC) subtypes depending on their characteristics: slow (MyHCI), oxidative (MyHCIIa), intermediate (MyHCIIx), and fast (MyHCIIb) [3]. The orchestration of the growth and maturation of skeletal muscle is finely tuned by a plethora of mediators and cascading signals. A growing body of evidence in recent years has elucidated the critical involvement of circRNA in orchestrating myogenic processes across diverse taxa [4,5,6]. The precise modes of action for many circRNAs remain to be unraveled fully, although it is expected that they may impact muscle genesis directly or indirectly. Therefore, comprehensive probing into numerous circRNAs is imperative for determining their role in skeletal muscle proliferation and maturation.

CircRNA forms a class of single-stranded non-coding RNA distinguished by its covalently sealed configuration. Recent progress in sequencing methodologies has enabled scientists to unearth a burgeoning repertoire of non-coding RNAs, using bioinformatics tools to identify numerous circRNAs in mammalian cells. CircRNA was found to exhibit enhanced conformational resilience and robustness against degradation by ribonuclease R (RNase R), showing a marked difference from its conventional linear RNA counterparts [7]. CircRNAs are ubiquitous across many cells and tissues, and they exert biological functions through multiple mechanisms, including serving as miRNA sponges, regulating RNA-binding proteins, and translating polypeptides [8,9,10]. For instance, circSGCB was found to inhibit myoblast proliferation while also promoting their differentiation through miR-27a-3p regulation [11]. CircACTA1 acts as a sponge for miR-433 and miR-199a-5p, contributing to the regulation of growth and development in bovine primary myoblasts [12]. Another circRNA, circMYBPC1, binds directly to MyHC protein, facilitating myoblast differentiation [13]. Similarly, circKANSL1L interacts with Akt through the encoded protein, leading to an enhanced transcriptional activity of FoxO3, which influences skeletal muscle generation [14]. Despite these recent strides, the intricate dynamics and multifaceted functionalities of circRNAs in skeletal muscle construction remain under-investigated, underscoring the need for intensified scrutiny into how they sculpt the landscape of muscular development.

Previous studies have demonstrated that high-throughput sequencing was conducted on the longissimus dorsi muscle of lean-type Large White pigs and obese-type Mashen pigs [15]. The results revealed significant differences in the expression of exonic circular RNA circAGGF1, which is derived from the AGGF1 gene, in the skeletal muscles of these two pig breeds. Therefore, we hypothesized that circAGGF1 may play a regulatory role in muscle development. Based on these observations, a comprehensive investigation of circAGGF1 as a candidate circular RNA was conducted. The critical role of circAGGF1 in skeletal muscle growth and development is underscored by this study, providing important insights for future research in this area.

## 2. Materials and Methods

### 2.1. Ethics Statement

All procedures on animals were approved by the Animal Care and Ethical Committee of Shanxi Agricultural University, China (SXAU-EAW-2024P.AA.012014236).

### 2.2. Cell Culture

The C2C12 cell line, sourced from Gaining Biological (Shanghai, China), was used for the experimental model. The growth medium was composed of 10% fetal bovine serum (Gibco, Waltham, MA, USA), 89% DMEM (Gibco, Waltham, MA, USA), and 1% penicillin-streptomycin (including 10,000 µg/mL of streptomycin and 10,000 U/mL of penicillin; Gibco, Waltham, MA, USA). When cell density attained 60–80% confluence, a differentiation medium made up of DMEM and 2% horse serum was employed to promote myoblast differentiation.

### 2.3. RNA Extraction and qRT-PCR

Total RNA extraction from tissue samples and cellular components was performed with TRIzol reagent (Thermo Fisher Scientific, Waltham, MA, USA), adhering rigorously to the procedure recommended by the vendor. Subsequent synthesis of complementary DNA (cDNA) was facilitated using the PrimeScript RT reagent Kit with gDNA Eraser (Takara, Japan) alongside the miRNA 1st Strand cDNA Synthesis Kit (by tailing A) (Vazyme, Nanjing, China). Quantitative reverse transcription PCR (qRT-PCR) analysis was executed with the AceQ Universal SYBR qPCR Master Mix (Vazyme, Nanjing, China). For normalization purposes during the assessment of mRNA, circRNA, and miRNA expressions, *GAPDH* and *U6* were engaged as endogenous control markers. Relative gene expression quantification was determined by applying the 2^−△△Ct^ method. Table 1 provides an inventory of all oligonucleotide primers used.

### 2.4. Immunofluorescence Staining

Following a thirty-minute stabilization in 4% paraformaldehyde, C2C12 cells were further treated with 0.5% Triton X-100 (Solarbio, Beijing, China) for another thirty minutes to facilitate membrane permeabilization. Upon completion of this step, the cells were incubated in 5% BSA for one hour prior to overnight incubation at 4 °C with the primary MyHC antibody (1:100, MF20, DSHB). Subsequent to the initial antibody binding phase, the cells were exposed to a fluorescent conjugated goat anti-mouse IgG secondary antibody, initiating a subsequent one-hour incubation carried out under ambient conditions shielded from light. Upon the conclusion of the secondary antibody interaction, DAPI staining was introduced, prompting an additional 15 min incubation at room temperature. The series of treatments culminated in the acquisition of visual data via an inverted fluorescence microscopy examination.

### 2.5. Luciferase Reporter Assay

A concentration of 1.5 × 10^4^ cells per well was seeded into 96-well plates using 293T cells. Successful plating was followed by transfection procedures, with the wells receiving a co-transfection mixture of either the PsiCHECK2-circAGGF1-WT construct combined with miR-199a-3p mimics, the aforementioned wild-type construct with negative control mimetics, the PsiCHECK2-circAGGF1-MUT with miR-199a-3p mimics, or the mutant variant with negative control mimics. Following a 48 h incubation phase, quantification of luminescence was carried out with the dual luciferase reporter assay kit (DL101-01, Vazyme), with the relative intensity of Renilla luciferase activity normalized against firefly luciferase as the reference standard.

### 2.6. Vector Construction and siRNA

The circAGGF1 overexpression vector (PCDNA3.1-circAGGF1) along with a control vector devoid of insert were constructed by Hanbio (Shanghai, China). The siRNA and NC of Fgf7 were custom-synthesized by Qingke (Beijing, China). Refer to Table 2 for an inventory of all the siRNA primers used. Cell transfections were executed employing Lipofectamine 3000 (Thermo Fisher Scientific, Waltham, MA, USA), adhering meticulously to the manufacturer’s guidelines.

### 2.7. Western Blot

Protein extraction from C2C12 cell cultures was facilitated with a RIPA buffer (BOSTER, Wuhan, China). The protein concentration was then determined with a BCA protein assay kit (Beyotime Biotechnology, Shanghai, China). Proteins were uniformly loaded onto a 10% SDS-PAGE gel before undergoing electrotransfer onto PVDF membranes (BOSTER, Wuhan, China). Optimal binding conditions were achieved by pre-treating the membranes with blocking buffer for 1 h before overnight immersion in primary antibodies MyoD (18943-1-AP, 1:1000, Proteintech, Wuhan, China), GAPDH (10494-1-AP, 1:5000, Proteintech, Wuhan, China), MyHCIIb (BF-F3, 1:200, DSHB, Lowa City, IA, USA), and MyHCI (BA-D5, 1:200, DSHB, USA). The membranes were then washed and probed with the secondary antibody (926-32211, LI-COR, Lincoln, NE, USA), after which they were exposed and photographed.

### 2.8. Animal Treatment and Establishment of Muscle Injury Animal Model

C57BL/6J male mice, aged 6 weeks and weighing 20.5 ± 0.4 g (n = 80), were obtained from Beijing SPF Biotechnology Co., Ltd. (Beijing, China). Of these, 20 mice were randomly assigned to two groups: the OE-circAGGF1 group and the OE-NC group, with 10 mice in each group. Mice in the OE-circAGGF1 group received 50 μL of adeno-associated virus overexpressing circAGGF1 (titer: 1.8 × 10^12^), while the OE-NC group received an equal volume of control adeno-associated virus. The remaining 60 mice were used to assess changes in skeletal muscle injury and were similarly divided into two groups: the OE-circAGGF1 group and the OE-NC group, with 30 mice in each group. Cardiotoxin (CTX) was dissolved in sterile saline to a final concentration of 10 μmol/L, and 50 μL was injected into the gastrocnemius (Gas) muscle of each mouse. At 5 and 14 days post-injury, 15 mice from each group were euthanized at each time point, and Gas muscle tissue samples were collected and fixed for hematoxylin-eosin (HE) staining.

### 2.9. Statistical Analysis

The results in our study have been summarized as the mean ± SD. SPSS 22.0 software was used for statistical analysis. Comparisons between the two experimental sets were assessed via Student’s *t*-test to ensure statistical robustness. ANOVA was used to analyze variances among three or more cohorts. Statistical significance was established at * *p* < 0.05 and ** *p* < 0.01.

## 3. Results

### 3.1. circAGGF1 Promotes Differentiation and Alters the Muscle Fiber Composition in C2C12 Cells

**Figure 1:**
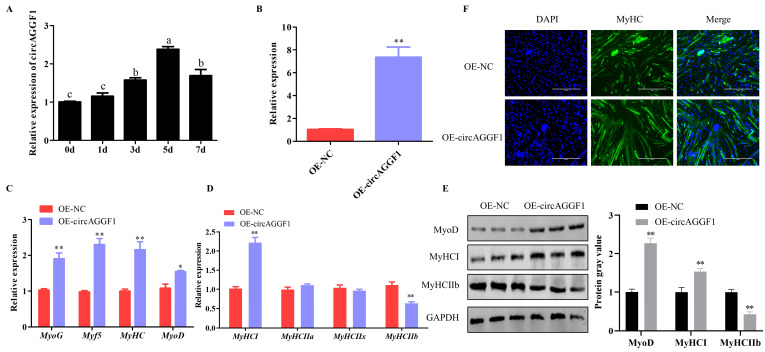
Effects of circAGGF1 on myogenesis of C2C12 cells. (**A**) CircAGGF1 expression patterns throughout cell differentiation. (**B**) CircAGGF1 transfection efficiency. (**C**,**D**) Myogenesis genes transcription in C2C12 cells following circAGGF1 overexpression. (**E**) Protein expression of myogenesis genes in C2C12 cells upon circAGGF1 overexpression. (**F**) Cell differentiation was assessed via MyHC and visualized using a fluorescence microscope. Scale bar represents 400 μm. * *p* < 0.05, ** *p* < 0.01. Different lowercase letters mean significant difference (*p* < 0.05).

In order to assess the influence of circAGGF1 on myoblast activity, we initially examined its expression profile during the differentiation of C2C12 cells. It was found that circAGGF1 expression increased as cell differentiation progressed (Figure 1A). The functional role of circAGGF1 was investigated further by transfecting C2C12 cells with a circAGGF1 overexpression vector, resulting in a transfection efficiency exceeding the baseline by a factor of six (Figure 1B), effectively paving the way for subsequent investigative assays. qRT-PCR and Western blot techniques were used to scrutinize the transcriptional profiles of myogenic marker genes. Strikingly, the results revealed that the enforced expression of circAGGF1 stimulated the mRNA expression of *MyoD*. Furthermore, significant increases in the mRNA levels of additional myogenic markers—*MyoG*, *Myf5*, and *MyHC*—were observed (Figure 1C). Meanwhile, it was found that the protein abundance of MyoD was notably elevated in the cohort subjected to circAGGF1 overexpression relative to the control group (Figure 1E). In addition, circAGGF1 augmentation resulted in a substantial elevation in the mRNA levels of *MyHCI* concurrently with a pronounced suppression of *MyHCIIb* within the cellular context of C2C12 (Figure 1D). The expression profiles of both of these proteins mirrored the corresponding mRNA expression trends (Figure 1E). To probe further into the effects of circAGGF1 on myotube morphogenesis, an immunofluorescence technique was applied. A pronounced enhancement in myotube maturation frequency was observed in the cohort subjected to circAGGF1 overexpression with respect to the control group (Figure 1F). Cumulatively, this body of evidence highlights the facilitative role of circAGGF1 in propelling the differentiation of C2C12 cells.

### 3.2. circAGGF1 Promotes Mice Myogenesis In Vivo

**Figure 2:**
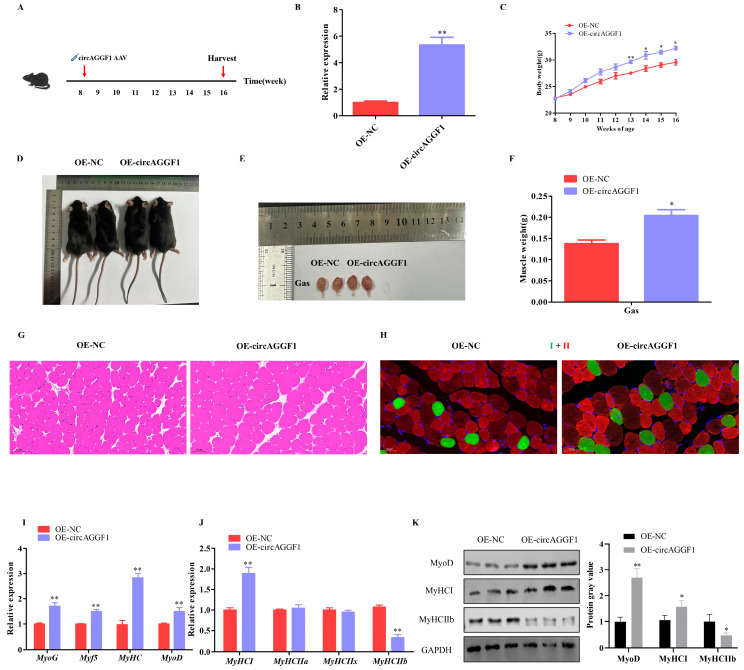
circAGGF1 Promotes mice myogenesis in vivo. (**A**) The animal experimental procedure. (**B**) Transfection efficiency of circAGGF1 in mouse muscle tissue. (**C**) Body weights progression in mice from 8 to 16 weeks old. (**D**) Gross morphology of mice in each group. (**E**,**F**) The gross morphology and weight of Gas muscle tissues. (**G**) HE staining. Scale bar indicates 50 μm. (**H**) Muscle fiber immunofluorescence. Green represents type I fibers, and red for type II fibers. Scale bar indicates 50 μm. (**I**–**K**) Expression of myogenesis genes at mRNA and protein in muscle tissue. * *p* < 0.05, ** *p* < 0.01.

CircAGGF1 adeno-associated virus was transfected into 8-week-old mice to achieve sustained in vivo overexpression of circAGGF1 (Figure 2A). The concentration of circAGGF1 in the muscular tissue from the OE-circAGGF1 group was much higher than the levels observed in the OE-NC cohort (Figure 2B). After 11 weeks, a conspicuous body mass disparity was measured, with the OE-circAGGF1 cluster exhibiting an increased average weight compared with the OE-NC group (Figure 2C,D). Moreover, a marked enhancement in Gas muscle mass was observed in the OE-circAGGF1 contingent as opposed to the OE-NC group (Figure 2E,F). These observations lend credence to the hypothesis that circAGGF1 can potentiate myogenic processes in rodent models in vivo.

Histological analysis through HE staining showcased a notable expansion in the cross-sectional areas (CSA) of myofibers within the OE-circAGGF1 cohort, surpassing measurements obtained from the OE-NC group (Figure 2G). Complementarily, immunofluorescence labeling unveiled an enhanced prevalence of type I myofiber density alongside a diminished count of type II fibers in the OE-circAGGF1 (Figure 2H). Furthermore, the overexpression of circAGGF1 was associated with enhanced mRNA expression of myogenic factors in mouse muscle tissues (Figure 2I). Precisely, there was a noticeable escalation in the mRNA and protein manifestations of MyHC I and MyoD, juxtaposed against a decrement in the mRNA and protein concentrations of MyHC IIb (Figure 2J,K). These collective outcomes allude to the pivotal function of circAGGF1 in advancing the process of myogenesis within living murine models.

**Figure 3:**
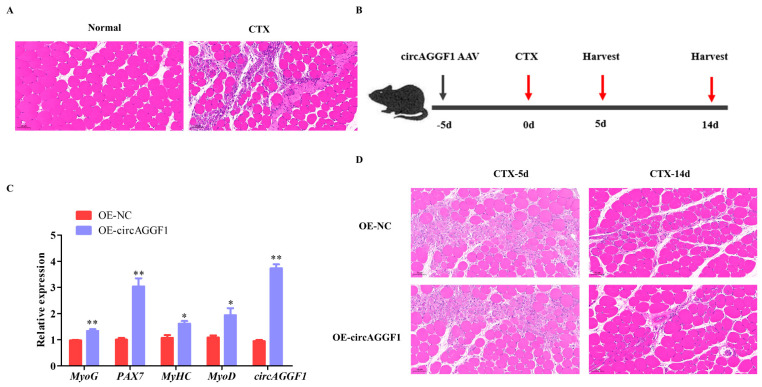
circAGGF1 enhances skeletal muscle regeneration. (**A**) HE staining of Gas muscles after injection of CTX. (**B**) The animal experimental procedure. (**C**) The expression of circAGGF1 and the changes of myogenesis genes in Gas muscle at 5 d, after CTX injury and AAV injection. (**D**) Following the transfection with circAGGF1, HE staining was performed on muscle 5 and 14 days after CTX injection. Scale bar indicates 50 μm. * *p* < 0.05, ** *p* < 0.01.

To investigate the potential regenerative capabilities enabled by circAGGF1 overexpression, cardiotoxin (CTX) was administered into the Gas muscle of the mice, inducing a controlled muscle damage scenario to enable the evaluation of subsequent repair mechanisms. HE staining revealed that the myofibers in the Gas muscle had undergone lysis, resulting in numerous scattered nuclei following treatment with CTX (Figure 3A), confirming the successful establishment of a muscle damage model in mice. Subsequently, prior to CTX injection, the mice were infected with an adenovirus-associated virus (AAV) that causes overexpression of circAGGF1 in order to maintain long-term circAGGF1 overexpression efficiency in vivo (Figure 3B). The Gas muscle was harvested for HE staining and qRT-PCR analysis at 5 and 14 days post-injury during muscle regeneration (Figure 3B), and a substantial boost in the mRNA concentrations of circAGGF1 was confirmed by day 5 post-treatment. Furthermore, amplified expressions of *MyoG*, *MyHC*, *MyoD*, and *PAX7* were observed in the overexpression group, with respect to the control counterparts (Figure 3C). HE staining revealed a reduction in muscle lysis in the circAGGF1 overexpression group at 5 days following CTX treatment; by 14 days, this cohort had developed an increased amount of new, smaller muscle bundles compared to the control (Figure 3D). These findings imply collectively that the overexpression of circAGGF1 promotes the recovery of Gas muscle following CTX-induced injury in an in vivo setting.

### 3.3. miR-199a-3p Inhibits Differentiation and Alters the Muscle Fiber Composition in C2C12 Cells

**Figure 4:**
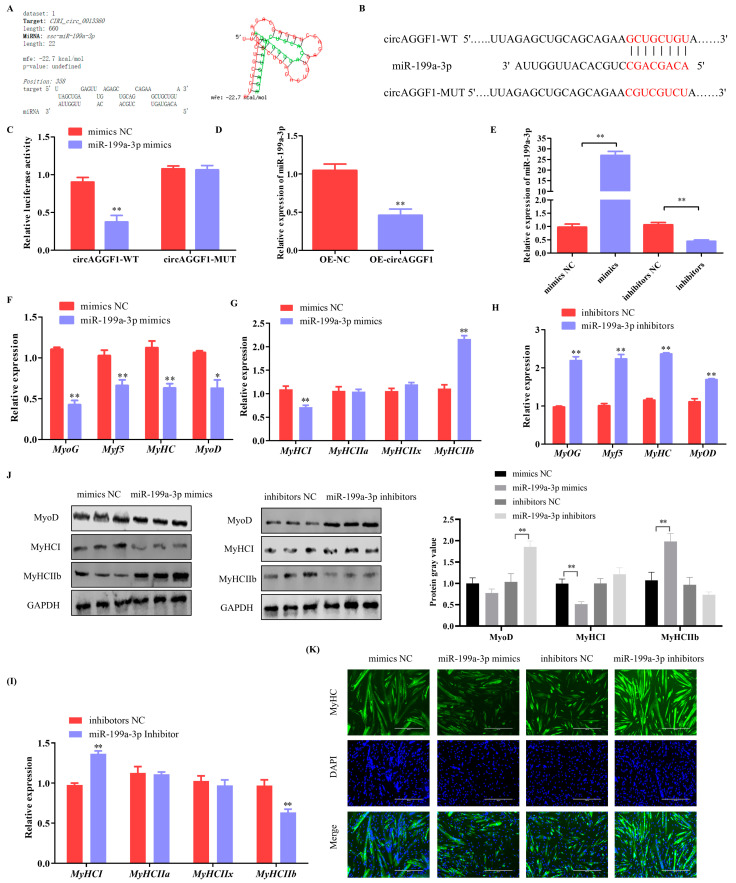
miR-199a-3p inhibits myogenesis in C2C12 Cells. (**A**) Predicted binding sites of circAGGF1 to miR-199a-3p using RNAhybrid software (http://bibiserv.techfak.uni-bielefeld.de/rnahybrid). (**B**) Schematic diagram of circAGGF1 wild-type and mutant luciferase vector construction. (**C**) Dual luciferase activity assay. (**D**) Effects of circAGGF1 on miR-199a-3p expression. (**E**) The miR-199a-3p overexpression and interference efficiency assay. (**F**,**G**) The mRNA expression of myogenesis genes in C2C12 cells that overexpress with miR-199a-3p. (**H**,**I**) The mRNA expression of myogenesis genes in C2C12 cells that Interference with miR-199a-3p. (**J**) Protein expression of MyoD, MyHCI, and MyHC IIb. (**K**) Results of circAGGF1 on myotube production. Scale bar indicates 400 μm. * *p* < 0.05, ** *p* < 0.01.

Bioinformatic scrutiny revealed putative interaction loci between miR-199a-3p and circAGGF1 (Figure 4A). A dual luciferase reporter construct was therefore fabricated and anchored around these specific docking points (Figure 4B). It was found that the introduction of miR-199a-3p mimics led to a notable suppression of the relative luminescent output of circAGGF1-WT, with no corresponding effect on circAGGF1-MUT (Figure 4C), suggesting that circAGGF1 can target and regulate miR-199a-3p. The amplification of circAGGF1 levels also reduced the expression profile of miR-199a-3p (Figure 4D). This evidence suggests that circAGGF1 operates as a ceRNA, effectively sponging miR-199a-3p.

To explore miR-199a-3p within the context of myogenic development, transfection experiments were performed using both a mimic and an inhibitor specific to miR-199a-3p on C2C12 cell cultures. The expression of miR-199a-3p upsurged considerably in cells treated with the mimic, whereas the inhibitor suppressed its expression markedly (Figure 4E). Cells exposed to the miR-199a-3p mimic also exhibited a pronounced decline in the expression profiles of canonical myogenic indicators (*MyHC*, *MyoG*, *Myf5*, and *MyoD*) (Figure 4F). Further examination revealed reduced *MyHCI* expression and a corresponding rise in *MyHCIIb* levels (Figure 4G). Western blot assays also showed a profound decrease in the protein levels of MyoD and MyHCI post-overexpression of miR-199a-3p along with an elevation in MyHCIIb protein expression (Figure 4J). Conversely, inhibition of miR-199a-3p yielded the opposite results (Figure 4H–J). Finally, immunofluorescence assays revealed an inhibition of myotube formation by miR-199a-3p (Figure 4K). In conclusion, miR-199a-3p serves as a negative regulator of myogenesis.

### 3.4. Fgf7 Promotes Differentiation and Alters the Muscle Fiber Composition in C2C12 Cells

**Figure 5:**
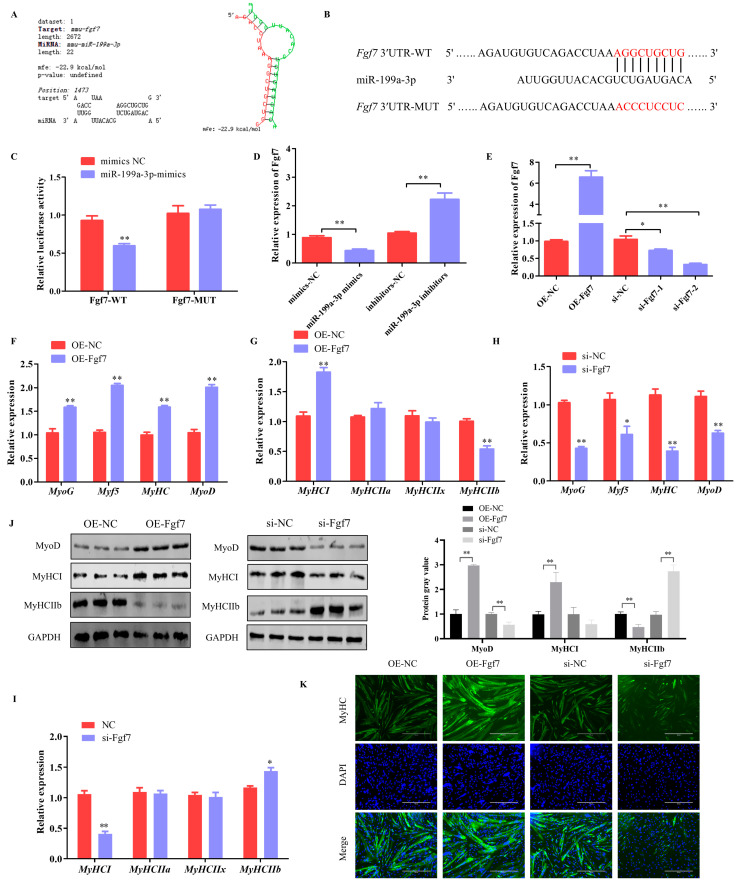
Fgf7 promotes myogenesis in C2C12 Cells. (**A**) Predicted binding sites of miR-199a-3p to Fgf7 using RNAhybrid software. (**B**) Schematic diagram of Fgf7 wild-type and mutant luciferase vector construction. (**C**) Dual luciferase activity assay. (**D**) Effects of miR-199a-3p on Fgf7 expression. (**E**) The Fgf7 overexpression and interference efficiency assay. (**F**,**G**) The mRNA expression of myogenesis genes in C2C12 cells that overexpress with Fgf7. (**H**,**I**) The mRNA expression of myogenesis genes in C2C12 cells that Interference with Fgf7. (**J**) Protein expression of MyoD, MyHCI, and MyHC IIb. (**K**) Results of Fgf7 on myotube production. Scale bar indicates 400 μm. * *p* < 0.05, ** *p* < 0.01.

Bioinformatics modeling revealed potential interaction zones between miR-199a-3p and Fgf7 (Figure 5A,B). These results suggested that administering miR-199a-3p mimics led to a notable suppression in the luminescent output associated with the Fgf7-WT reporter construct, while the mutated version displayed negligible alterations (Figure 5C). This observation implies that miR-199a-3p can target and modulate Fgf7. Additionally, the expression level of Fgf7 was modulated by miR-199a-3p (Figure 5D). These results suggest the direct influence on Fgf7 by miR-199a-3p.

The function of Fgf7 on myogenic progression was investigated via transfection experiments involving both the overexpression and knockdown of Fgf7 in C2C12 cell lines. A high efficacy of Fgf7 manipulation was observed in the quantitative assessments (Figure 5E). Critically, the enforced upregulation of Fgf7 prompted a discernible surge in the expression of known myogenic markers (*MyoD*, *Myf5*, *MyHC*, and *MyoG*) (Figure 5F). Furthermore, these observations highlighted an enhancement in *MyHCI* and a corresponding diminution in *MyHCIIb* expression (Figure 5G). Western blotting further confirmed a significant increase in MyoD and MyHCI protein levels after the overexpression of Fgf7, alongside a marked decrease in the protein levels of MyHCIIb (Figure 5J). Conversely, the inhibition of Fgf7 elicited the opposite response (Figure 5H–J). Furthermore, it was revealed by immunofluorescence assays that Fgf7 enhances myotube formation (Figure 5K). In summary, these results demonstrate that Fgf7 can promote the myogenesis of C2C12 cells.

### 3.5. CircAGGF1 Promotes Myoblast Differentiation and Myofiber Type Transformation via miR-199a-3p

**Figure 6:**
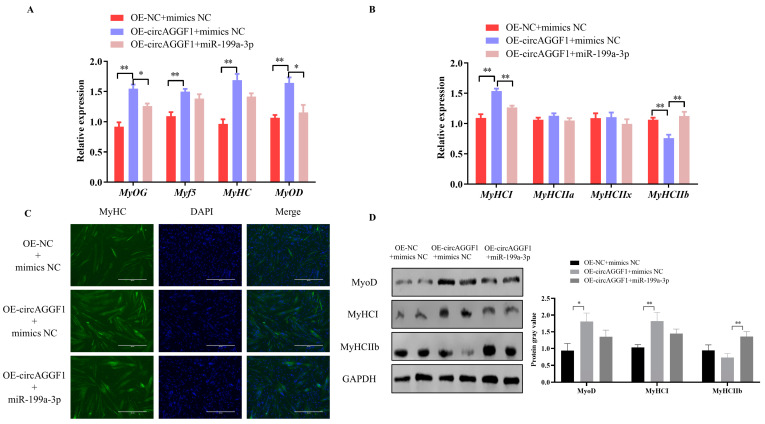
circAGGF1 influences myoblast differentiation and the transformation of myofiber types in C2C12 cells via miR-199a-3p. (**A**) Expression changes of key myogenic factors after transfection with OE-NC + mimics NC, OE-circAGGF1 + mimics NC, and OE-circAGGF1+ miR-199a-3p. (**B**) Expression changes of key myofiber-type factors after transfection with OE-NC + mimics NC, OE-circAGGF1 + mimics NC, and OE-circAGGF1 + miR-199a-3p. (**C**) Immunofluorescence staining results after transfection with OE-NC + mimics NC, OE-circAGGF1 + mimics NC, and OE-circAGGF1 + miR-199a-3p. Blue indicates nuclei stained with DAPI; green indicates MyHC protein. Scale bar indicates 400 μm. (**D**) Protein expression of MyoD, MyHCI, and MyHC IIb. * *p* < 0.05, ** *p* < 0.01.

**Figure 7:**
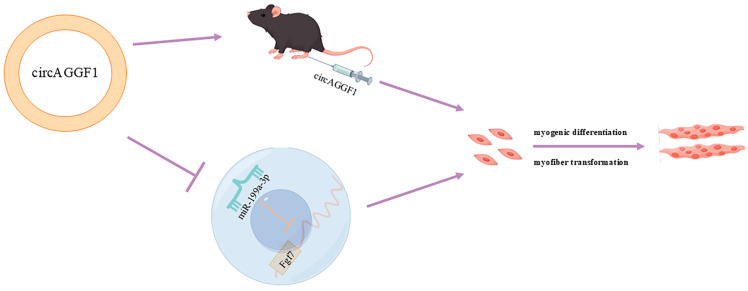
Mechanism of circAGGF1 in regulating myogenesis.

Building upon the established interplay between circAGGF1 and miR-199a-3p, recuperation trials were conducted through co-transfecting the OE-circAGGF1 plasmid alongside miR-199a-3p mimics into C2C12 muscle cell cultures in order to investigate whether the amplified expression of miR-199a-3p could counteract the influence of circAGGF1. It was revealed that the augmentation of circAGGF1 expression elevated the mRNA and protein expressions of genes involved in cellular differentiation, while concurrent transfection featuring miR-199a-3p induced a corresponding decline (Figure 6A,D). Moreover, the overexpression of circAGGF1 elevated both the mRNA and protein expressions of the porcine muscle fiber gene MyHCI, while simultaneously reducing the levels of MyHCIIb at both protein stages and in mRNA. Conversely, co-transfection with miR-199a-3p suppressed the expression profiles of these genes (Figure 6B,D). Immunofluorescence analysis confirmed that the overexpression of miR-199a-3p negated the stimulatory effect of circAGGF1 on MyHC expression and on the process of myotube formation (Figure 6C). It can therefore be deduced that miR-199a-3p acts in opposition to circAGGF1 on myogenesis, exerting an influence on myofiber type transformation within C2C12 cells. In conclusion, this study demonstrates the promotion of myogenesis in vitro and in vivo by circAGGF1. Finally, a new mechanism for regulating myogenesis, circAGGF1/miR-199a-3p/Fgf7, has been identified (Figure 7).

## 4. Discussion

Myogenesis is a multifaceted biological undertaking involving the expansion and specialization of myoblasts and the genesis of myotubes, modulated by a plethora of myogenic regulatory elements [16,17]. The specific role of circRNAs in this process remains inadequately understood. The C2C12 cell line has been extensively employed as an important in vitro model for investigations of muscle metabolism and differentiation. Upon being cultured in a differentiation-inducing medium, C2C12 cells fuse rapidly, forming multinucleated myotubes [18]. They therefore provide a crucial milieu for probing the intricacies of skeletal muscle metabolism and differentiation at both the cellular and molecular level [19]. Previous studies have revealed the expression of circAGGF1 at high levels in pig skeletal muscle; based on this work, the potential role of circAGGF1 in the molecular mechanisms underlying myogenic differentiation has been investigated.

It was noted in this study that the initial rise in circAGGF1 expression was followed by a decremental phase associated with myoblast differentiation, hinting at the potential pivotal involvement of circAGGF1 in the orchestration of myoblast differentiation processes. This hypothesis was validated further by investigations of the specific role played by circAGGF1 in myogenesis within myoblasts. The present study has revealed the dual role of circAGGF1 in myoblast differentiation, promoting both this process and the concurrent expression and inhibition of MyHCI and MyHCIIb, respectively. Additionally, in vivo assays performed within murine models unveiled the capability of circAGGF1 to catalyze skeletal muscle advancement and to augment the ratio of type I muscle fibers, which are vital components for sustaining physical endurance and corporal equilibrium that contract slowly and can sustain prolonged activity without significant fatigue [20]. These fibers, rich in mitochondria, are the primary sites for aerobic metabolism. By using oxygen to oxidize fats and glycogen, they produce ATP as a continuous source of energy for muscle contraction [21]. Therefore, type I muscle fibers possess a high oxidative metabolic capacity. Previous studies showed that circDdb1 can bind to eEF2 by encoding a novel protein, circDdb1-867aa. This increases the level of phosphorylation, mitigating protein translation and promoting muscle atrophy [22]. Concurrently, FoxOs modulate the transcription of genes implicated in the wasting of skeletal muscle (including MAFbx and MuRF1), along with genes related to autophagy, via twin proteolysis routes: the ALP and UPS [23,24,25,26]. The overexpression of circTmeff1 prompts muscle atrophy, mediated by direct interaction with TDP-43 and the encoding of a protein, TMEFF1-339aa [27]. It was demonstrated that circFUT10 regulates the regenerative potential of aged skeletal muscle stem cells through the targeting of HOXA9. CircFUT10 overexpression promotes self-renewal and muscle regeneration in aged stem cells, whereas its inhibition significantly impairs cell proliferation and reduces regenerative capacity [28]. The present study has further illustrated that circAGGF1 can facilitate recovery and regeneration following skeletal muscle injury in mice.

Previous research has verified that circRNAs can cause a notable alteration in the expression of their target genes by competitively binding to miRNAs [29,30,31,32,33]. A combination of qRT-PCR and bioinformatics analysis results, described in this study, has led to a preliminary speculated correlation between circAGGF1 and miR-199a-3p, further corroborated by dual-luciferase reporter assays. Preceding studies showed the intimate connection between miR-199a-3p and the multiplication and programmed cell death of a range of cellular entities [34,35]; although its contribution to skeletal muscle expansion and maturation is still largely uncharted territory. This investigation has illustrated the capacity of miR-199a-3p to suppress myogenic differentiation in myoblast cells, to diminish the manifestation of MyHCI, and concomitantly to amplify the expression levels of MyHCIIb. Bioinformatics methods were used to analyze the potential downstream target genes of miR-199a-3p, confirming Fgf7 as a target through luciferase reporter gene detection and qRT-PCR analysis. The repression of Fgf7 by miR-199a-3p was revealed, underscoring its pivotal function in negatively modulating Fgf7. Fgf7, known alternatively as keratinocyte growth factor (KGF), is a notable component in the FGF family [36,37,38,39], which also promotes the proliferation of epithelial cells and protects against apoptosis upon binding to its receptor [40,41]. Fgf7 enhances the integration of islets in the liver, contributing to the normalization of blood glucose levels while also maintaining a favorable safety profile [42]. This research has also revealed that Fgf7 can augment the myogenic transformation of myoblasts while also upregulating the expression of type I muscle fibers. Further investigations have demonstrated that Fgf7 catalyzes the proliferation of myoblasts, accelerating the regeneration of skeletal muscle post-damage in murine models [43]. The biological roles of circAGGF1 mediated via miR-199a-3p were studied further with co-transfection assays executed in a C2C12 cellular environment, in which OE-circAGGF1 was introduced in conjunction with miR-199a-3p analogues. The results indicated that OE-circAGGF1 plays a significant role in enhancing myogenesis in myoblasts, whereas the opposite effect was observed with the overexpression of miR-199a-3p. Collectively, these findings suggest that circAGGF1 mitigates its inhibitory impact on the target gene (Fgf7) by binding competitively to miR-199a-3p, facilitating the promotion of myogenesis in myoblasts.

## 5. Conclusions

This study has revealed that circAGGF1 acts as a molecular decoy for miR-199a-3p, thereby facilitating myogenic progression in C2C12 cellular environments. Furthermore, circAGGF1 can enhance the recovery and regeneration of mouse skeletal muscle following injury. This research furthers our understanding of the regulatory mechanisms orchestrating myogenic advancement, laying the fundamental groundwork for deeper explorations into the intricate underpinnings of myogenesis.

## Figures and Tables

**Table 1:** The primers.

Primer Names	Primers Sequences (5′→3′)	Annealing Temperature (°C)	Product Length (bp)
circAGGF1	F: ACTGTGATGTGGAAAGTGGTCG R: CTTCGTTTCTCCCACAATGGAG	59	189
*MyoD*	F: TGCTCTGATGGCATGATGGATT R: AGATGCGCTCCACTATGCTG	60	163
*MyoG*	F: GTCCCAACCCAGGAGATCAT R: CACGATGGACGTAAGGGAG	58	77
*Myf5*	F: CTCTGACGGCATGCCTGAAT R: AGCTCGGATGGCTCTGTAG	58	177
*MyHC*	F:GGATGGGATATAAAGGGGCTGG R: ATCCAGAGATCCTGGGTTGGA	59	72
*MyHCIIx*	F: GCATCCCTAAAGGCAGGCT R: GTTCTGAGCCTCGATTCGCT	60	139
*MyHCIIa*	F:AGGAGCTTACTTACCAGACAGA R: TCGCTTCGGTCATTCCACAG	57	299
*MyHCIIb*	F: CTCACCTACCAGACCGAGGA R: CTCCTGTCACCTCTCAACAGA	59	290
*MyHCI*	F: AGGTCTGGCTCTGAGCATTC R: CCTTTCTCGGAGCCACCTTG	60	244
*GAPDH*	F: CTTCTCCTGCAGCCTCGT R: ATGAAGGGGTCGTTGATGGC	59	137
*U6*	F: CTCGCTTCGGCAGCACA R: AACGCTTCACGAATTTGCGT	60	185
*PAX7*	F: GAGAACCCCGGGATGTTCAG R: ATCGAACTCACTGAGGGCAC	60	92
miR-199a-3p	GCGCGACAGTAGTCTGCACAT		
*Fgf7*	F: TATTCATGAACACCCGGGGC R: CAGTTCACACTCGTAGCCGT	60	168

**Table 2:** The vector sequence.

Names	Sequences (5′→3′)
miR-199a-3p mimics	F: ACAGUAGUCUGCACAUUGGUUAACC R: AAUGUGCAGACUACUGUUU
miR-199a-3p inhibitor	UAACCAAUGUGCAGACUACUGU
si-Fgf7-1	CACCUAUGCAUCAGCUAAATT
si-Fgf7-2	GGUACCUGAGGAUUGACAATT

## Data Availability

None of the data were deposited in an official repository. The data are available from the authors upon request.

## References

[1] Dayanidhi S., Lieber R.L. (2014). Skeletal muscle satellite cells: Mediators of muscle growth during development and implications for developmental disorders. Muscle Nerve.

[2] Kopantseva E.E., Belyavsky A.V. (2016). [Key regulators of skeletal myogenesis]. Mol. Biol..

[3] Schiaffino S., Reggiani C. (2011). Fiber types in mammalian skeletal muscles. Physiol. Rev..

[4] Han S., Cui C., Wang Y., He H., Liu Z., Shen X., Chen Y., Li D., Zhu Q., Yin H. (2019). Knockdown of CSRP3 inhibits differentiation of chicken satellite cells by promoting TGF-β/Smad3 signaling. Gene.

[5] Liang G., Yang Y., Niu G., Tang Z., Li K. (2017). Genome-wide profiling of Sus scrofa circular RNAs across nine organs and three developmental stages. DNA Res..

[6] Ouyang H., Chen X., Wang Z., Yu J., Jia X., Li Z., Luo W., Abdalla B.A., Jebessa E., Nie Q. (2018). Circular RNAs are abundant and dynamically expressed during embryonic muscle development in chickens. DNA Res..

[7] Chen W., Schuman E. (2016). Circular RNAs in Brain and Other Tissues: A Functional Enigma. Trends Neurosci..

[8] Zheng S., Zhang X., Odame E., Xu X., Chen Y., Ye J., Zhou H., Dai D., Kyei B., Zhan S. (2021). CircRNA-Protein Interactions in Muscle Development and Diseases. Int. J. Mol. Sci..

[9] Li L., Chen Y., Nie L., Ding X., Zhang X., Zhao W., Xu X., Kyei B., Dai D., Zhan S. (2019). MyoD-induced circular RNA CDR1as promotes myogenic differentiation of skeletal muscle satellite cells. Biochim. Biophys. Acta Gene Regul. Mech..

[10] Shi Y., Jia X., Xu J. (2020). The new function of circRNA: Translation. Clin. Transl. Oncol..

[11] Xu J., Wen Y., Li X., Peng W., Zhang Z., Liu X., Yang P., Chen N., Lei C., Zhang J. (2024). Bovine enhancer-regulated circSGCB acts as a ceRNA to regulate skeletal muscle development via enhancing KLF3 expression. Int. J. Biol. Macromol..

[12] Qi A., Ru W., Yang H., Yang Y., Tang J., Yang S., Lan X., Lei C., Sun X., Chen H. (2022). Circular RNA ACTA1 Acts as a Sponge for miR-199a-5p and miR-433 to Regulate Bovine Myoblast Development through the MAP3K11/MAP2K7/JNK Pathway. J. Agric. Food Chem..

[13] Chen M., Wei X., Song M., Jiang R., Huang K., Deng Y., Liu Q., Shi D., Li H. (2021). Circular RNA circMYBPC1 promotes skeletal muscle differentiation by targeting MyHC. Mol. Therapy. Nucleic Acids.

[14] Lin Z., Xie F., He X., Wang J., Luo J., Chen T., Jiang Q., Xi Q., Zhang Y., Sun J. (2024). A novel protein encoded by circKANSL1L regulates skeletal myogenesis via the Akt-FoxO3 signaling axis. Int. J. Biol. Macromol..

[15] Li M., Zhang N., Zhang W., Hei W., Cai C., Yang Y., Lu C., Gao P., Guo X., Cao G. (2021). Comprehensive analysis of differentially expressed circRNAs and ceRNA regulatory network in porcine skeletal muscle. BMC Genom..

[16] Mohammadabadi M., Bordbar F., Jensen J., Du M., Guo W. (2021). Key Genes Regulating Skeletal Muscle Development and Growth in Farm Animals. Animals.

[17] Weskamp K., Olwin B.B., Parker R. (2021). Post-Transcriptional Regulation in Skeletal Muscle Development, Repair, and Disease. Trends Mol. Med..

[18] Ding H., Heng B., He W., Shi L., Lai C., Xiao L., Ren H., Mo S., Su Z. (2016). Chronic reactive oxygen species exposure inhibits glucose uptake and causes insulin resistance in C2C12 myotubes. Biochem. Biophys. Res. Commun..

[19] Wong C.Y., Al-Salami H., Dass C.R. (2020). C2C12 cell model: Its role in understanding of insulin resistance at the molecular level and pharmaceutical development at the preclinical stage. J. Pharm. Pharmacol..

[20] Nezhad F.Y., Riermeier A., Schönfelder M., Becker L., de Angelis M.H., Wackerhage H. (2022). Skeletal muscle phenotyping of Hippo gene-mutated mice reveals that Lats1 deletion increases the percentage of type I muscle fibers. Transgenic Res..

[21] Plotkin D.L., Roberts M.D., Haun C.T., Schoenfeld B.J. (2021). Muscle Fiber Type Transitions with Exercise Training: Shifting Perspectives. Sports.

[22] Zhu X., Yang T., Zheng Y., Nie Q., Chen J., Li Q., Ren X., Yin X., Wang S., Yan Y. (2024). EIF4A3-Induced Circular RNA CircDdb1 Promotes Muscle Atrophy through Encoding a Novel Protein CircDdb1-867aa. Adv. Sci..

[23] Huang Z., Fang Q., Ma W., Zhang Q., Qiu J., Gu X., Yang H., Sun H. (2019). Skeletal Muscle Atrophy Was Alleviated by Salidroside Through Suppressing Oxidative Stress and Inflammation During Denervation. Front. Pharmacol..

[24] Bodine S.C., Latres E., Baumhueter S., Lai V.K., Nunez L., Clarke B.A., Poueymirou W.T., Panaro F.J., Na E., Dharmarajan K. (2001). Identification of ubiquitin ligases required for skeletal muscle atrophy. Science.

[25] Mammucari C., Milan G., Romanello V., Masiero E., Rudolf R., Del Piccolo P., Burden S.J., Di Lisi R., Sandri C., Zhao J. (2007). FoxO3 controls autophagy in skeletal muscle in vivo. Cell Metab..

[26] Zhao J., Brault J.J., Schild A., Cao P., Sandri M., Schiaffino S., Lecker S.H., Goldberg A.L. (2007). FoxO3 coordinately activates protein degradation by the autophagic/lysosomal and proteasomal pathways in atrophying muscle cells. Cell Metab..

[27] Chen R., Yang T., Jin B., Xu W., Yan Y., Wood N., Lehmann H.I., Wang S., Zhu X., Yuan W. (2023). CircTmeff1 Promotes Muscle Atrophy by Interacting with TDP-43 and Encoding A Novel TMEFF1-339aa Protein. Adv. Sci..

[28] Zhu M., Lian C., Chen G., Zou P., Qin B.G. (2021). CircRNA FUT10 regulates the regenerative potential of aged skeletal muscle stem cells by targeting HOXA9. Aging.

[29] Cao J., Zhang X., Xu P., Wang H., Wang S., Zhang L., Li Z., Xie L., Sun G., Xia Y. (2021). Circular RNA circLMO7 acts as a microRNA-30a-3p sponge to promote gastric cancer progression via the WNT2/β-catenin pathway. J. Exp. Clin. Cancer Res..

[30] Li H., Wei X., Yang J., Dong D., Hao D., Huang Y., Lan X., Plath M., Lei C., Ma Y. (2018). circFGFR4 Promotes Differentiation of Myoblasts via Binding miR-107 to Relieve Its Inhibition of Wnt3a. Mol. Therapy. Nucleic Acids.

[31] Ebbesen K.K., Kjems J., Hansen T.B. (2016). Circular RNAs: Identification, biogenesis and function. Biochim. Biophys. Acta.

[32] Li H., Zhang H., Dai Y., Li S., Gu J., Wu R., Jia J., Shen J., Zhang Y., Li H. (2024). CircITGB5 regulates the proliferation and adipogenic differentiation of chicken intramuscular preadipocytes through the miR-181b-5p/CPT1A axis. Int. J. Biol. Macromol..

[33] Zhang Z., Fan Y., Deng K., Liang Y., Zhang G., Gao X., El-Samahy M.A., Zhang Y., Deng M., Wang F. (2022). Circular RNA circUSP13 sponges miR-29c to promote differentiation and inhibit apoptosis of goat myoblasts by targeting IGF1. FASEB J..

[34] Wu S., Shao T., Xie J., Li J., Sun L., Zhang Y., Zhao L., Wang L., Li X., Zhang L. (2024). MiR-199a-3p regulates HCT-8 cell autophagy and apoptosis in response to Cryptosporidium parvum infection by targeting MTOR. Commun. Biol..

[35] Zhuo L., Zhou Y., Tian J., Li Y., Xie Z., Pei C., Yan B., Ma L. (2023). The role of miR-199a-3p in inhibiting the proliferation of spermatogonial stem cells under heat stress. Theriogenology.

[36] Wang L., Zhang X., Li H., Mou Y., Cui G. (2024). SP1 promotes high glucose-induced lens epithelial cell viability, migration and epithelial-mesenchymal transition via regulating FGF7 and PI3K/AKT pathway. Int. Ophthalmol..

[37] Fu W., Liu L., Tong S. (2024). Berberine inhibits the progression of breast cancer by regulating METTL3-mediated m6A modification of FGF7 mRNA. Thorac. Cancer.

[38] Zheng Y., Liu W.H., Yang B., Milman Krentsis I. (2024). Primer on fibroblast growth factor 7 (FGF 7). Differentiation.

[39] Zinkle A., Mohammadi M. (2019). Structural Biology of the FGF7 Subfamily. Front. Genet..

[40] Forbes S.J., Themis M., Alison M.R., Sarosi I., Coutelle C., Hodgson H.J. (2000). Synergistic growth factors enhance rat liver proliferation and enable retroviral gene transfer via a peripheral vein. Gastroenterology.

[41] Takase H.M., Itoh T., Ino S., Wang T., Koji T., Akira S., Takikawa Y., Miyajima A. (2013). FGF7 is a functional niche signal required for stimulation of adult liver progenitor cells that support liver regeneration. Genes Dev..

[42] Alwahsh S.M., Qutachi O., Starkey Lewis P.J., Bond A., Noble J., Burgoyne P., Morton N., Carter R., Mann J., Ferreira-Gonzalez S. (2021). Fibroblast growth factor 7 releasing particles enhance islet engraftment and improve metabolic control following islet transplantation in mice with diabetes. Am. J. Transplant..

[43] Ma L., Meng Y., An Y., Han P., Zhang C., Yue Y., Wen C., Shi X., Jin J., Yang G. (2024). Single-cell RNA-seq reveals novel interaction between muscle satellite cells and fibro-adipogenic progenitors mediated with FGF7 signalling. J. Cachexia Sarcopenia Muscle.

